# Invasive pneumococcal disease by serotype 23B in an asplenic unvaccinated patient: A case report

**DOI:** 10.1016/j.idcr.2026.e02698

**Published:** 2026-08-04

**Authors:** Yudai Tanaka, Koji Hayashi, Yuki Abe, Sayaka Uesaka, Hiroaki Maeda, Asuka Suzuki, Yuka Nakaya, Yoshihiro Takamura, Mamiko Sato, Yasutaka Kobayashi, Ippei Sakamaki

**Affiliations:** a Department of Infectious Diseases, Faculty of Medical Sciences, University of Fukui, Fukui, Japan; b Department of Rehabilitation Medicine, Fukui General Hospital, Fukui, Japan; c Department of Ophthalmology, Faculty of Medical Sciences, University of Fukui, Japan; d Department of Ophthalmology, Fukui General Hospital, Fukui, Japan; e Graduate School of Health Science, Fukui Health Science University, Fukui, Japan

**Keywords:** invasive pneumococcal disease, Streptococcus pneumoniae, serotype 23B, PCV21

## Abstract

Invasive pneumococcal disease (IPD) is a life-threatening infection caused by *Streptococcus pneumoniae*, particularly in immunocompromised individuals, such as those with asplenia. Although pneumococcal vaccination has reduced the incidence of vaccine-covered serotypes, non-vaccine serotypes, including serotype 23B, have emerged as the predominant cause of IPD. This case report describes a 67-year-old woman with asplenia who developed IPD　caused by multidrug-resistant Streptococcus pneumoniae serotype 23B, which led to meningitis, septic arthritis, and endophthalmitis. The patient, who had declined pneumococcal vaccination after her splenectomy, presented with fever, pain in her left hip and right shoulder, and visual issues. The diagnosis confirmed S. pneumoniae serotype 23B, resistant to multiple antibiotics. The treatment involved a combination of vancomycin, ceftriaxone, and rifampicin, followed by vitrectomy for the eye infection. Despite severe complications, including worsening vision, the patient’s vision in the right eye improved after surgery, and the joint pain subsided with long-term antibiotic therapy. This case underscores the challenges posed by non-vaccine *S. pneumoniae* serotypes, particularly in asplenic patients. Currently, we can use the 21-valent pneumococcal conjugate vaccine (PCV21), which includes the 23B serotype. Vaccination and early recognition, combined with a multidisciplinary treatment approach involving both antimicrobial therapy and surgical intervention, are crucial for improving the outcomes of patients with severe IPD.

## Highlights

• We report an invasive pneumococcal case caused by a multidrug-resistant serotype 23B.
• Early combination antimicrobial therapy and surgery enabled survival and visual recovery.
• It is very important for high-risk patients to receive the new vaccine.

## Introduction

Invasive pneumococcal disease (IPD) is a severe and often life-threatening infection caused by *Streptococcus pneumoniae*, a gram-positive bacterium responsible for a wide array of infections, including bacteremia, pneumonia, meningitis, and septic arthritis. IPD remains a major global health concern owing to its high morbidity and mortality, especially in vulnerable populations, such as the elderly, individuals with chronic diseases, and those with compromised immune systems. One of the most at-risk groups includes patients who have undergone splenectomy and those with congenital or acquired asplenia. The spleen plays a crucial role in the immune response by filtering the blood, removing pathogens, and playing a pivotal role in the opsonization of encapsulated bacteria. Thus, individuals without a spleen have an impaired immune response, particularly to encapsulated organisms such as *S. pneumoniae*, which increases their susceptibility to invasive infections, including severe forms such as meningitis and bacteremia.

The introduction of pneumococcal vaccines has significantly reduced the incidence of IPD caused by vaccine-covered serotypes, particularly in children and the elderly [1], [2]. The 23-valent pneumococcal polysaccharide vaccine (PPV23) and 13-valent pneumococcal conjugate vaccine (PCV13) have been instrumental in decreasing the prevalence of IPD caused by common serotypes, such as 1, 3, 4, and 14, included in the vaccines. Subsequently, broader-valency conjugate vaccines, including PCV20, have been recommended to expand the serotype coverage. However, despite these efforts, an emerging threat is the rise of non-vaccine serotypes. These non-vaccine serotypes have increasingly become the leading cause of IPD [3], [4], [5]. More recently, PCV 21 has become available and includes several serotypes that were not covered by earlier pneumococcal vaccines, it is recommended as an option for adults for whom pneumococcal conjugate vaccination is including immunocompromised individuals.

Non-vaccine serotypes, such as *S. pneumoniae* serotype 23B, often exhibit higher levels of antimicrobial resistance than their vaccine-covered counterparts, further complicating treatment strategies [6]. This is of particular concern because these strains demonstrate resistance to commonly used antibiotics, such as penicillin, cephalosporins, and macrolides. This antimicrobial resistance has been attributed to the overuse and misuse of antibiotics, which has led to selective pressure favoring resistant strains. Furthermore, IPD caused by multidrug-resistant (MDR) pneumococci is difficult to treat, particularly in immunocompromised individuals. MDR pneumococci are defined as pneumococci resistant to three or more antimicrobials classes [7]. In some cases, combination antimicrobial therapy and surgical intervention are required. The emergence of resistant serotypes poses a significant challenge to clinicians, who must continually adapt treatment protocols to account for these evolving pathogens.

The increasing prevalence of non-vaccine serotypes and the growing problem of antimicrobial resistance necessitate enhanced surveillance and vigilance. Furthermore, it highlights the need for updated treatment strategies and continued research into the evolving landscape of pneumococcal infections, particularly those caused by non-vaccine serotypes. Clinicians must be aware of the risks posed by non-vaccine serotypes and adapt their treatment approaches to ensure the best possible outcomes for patients, particularly those with compromised immune systems. It is important for clinicians to consider the role of combination antibiotic therapy and the need for surgical options.

## Case presentation

**Fig. 1:**
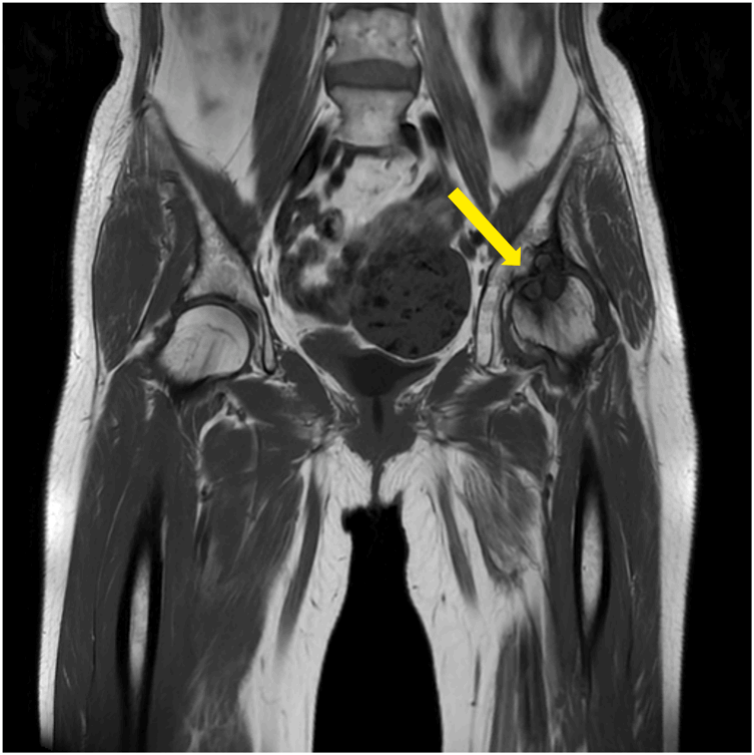
Magnetic resonance imaging (MRI) of the left hip. An MRI scan at the time of presentation revealed a low-signal area on T2 weighted images of the left femoral head and labrum, suggesting cystic changes. These findings are consistent with the osteoarthritic changes caused by congenital hip dislocations.

**Fig. 2:**
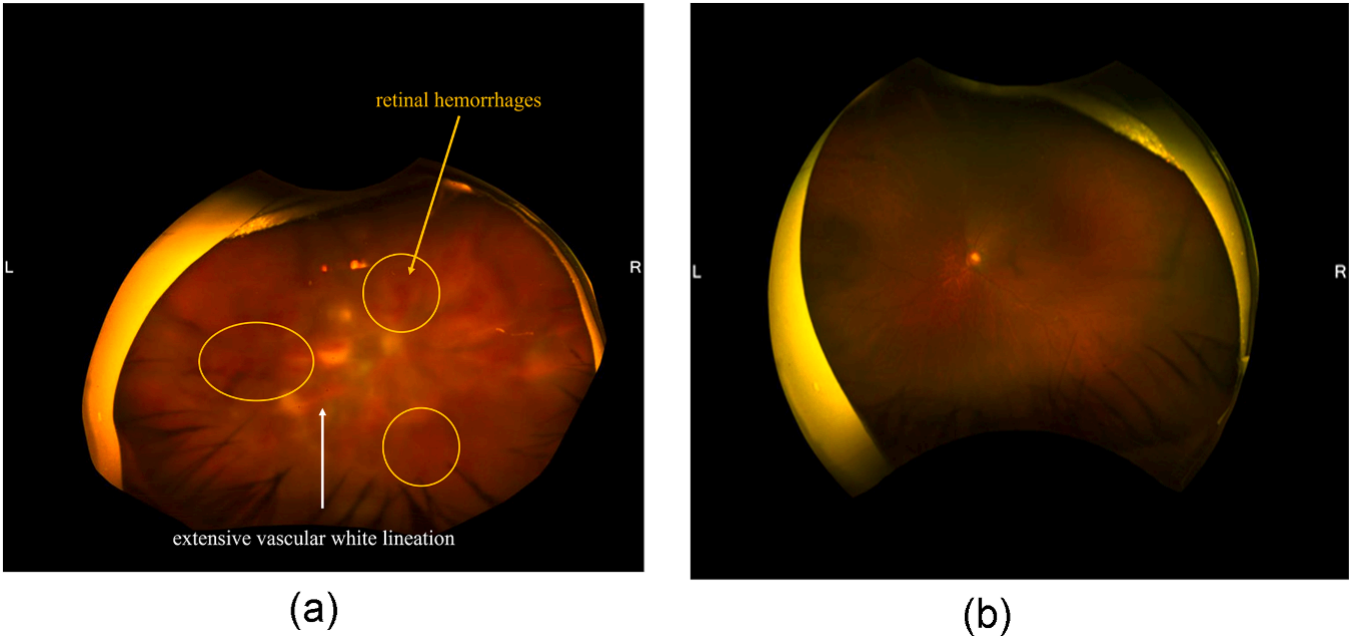
Fundus photographs on postoperative day 3. Fundus photograph of the right eye showing extensive vascular white lineation and retinal hemorrhage, indicating a poor visual prognosis (a). Fundus photograph of the left eye showing no signs of infection (b).

A 67-year-old Japanese woman with a history of osteoarthritis related to congenital hip dislocation (scheduled for elective surgery) and splenectomy at age 55 developed fever, followed by pain in her left hip and right shoulder. She had no history of vaccination against pneumococcal, Haemophilus influenzae type B, or meningococcal infections. She visited the Department of Orthopedics, where she received symptomatic treatment. The following day, her symptoms worsened, with new chills and inability to move, and she was transported to our hospital. Vital signs on admission showed an E3V4M6 on the Glasgow Coma Scale, body temperature of 37.8 °C, blood pressure of 146/75 mmHg, pulse rate of 76 bpm, and oxygen saturation of 97%. Neck stiffness was unremarkable; however, floaters in the right eye, slight headache, and pain when flexing the hip were noted. Blood tests revealed elevated levels of white blood cells (18.8 ×103/mL), glucose (130 mg/dL), urea nitrogen (46.6 mg/dL), creatinine (1.13 mg/dL), lactate dehydrogenase (253 70 U/L), and C-reactive protein (28.49 mg/dL). There were also decreased levels of albumin (4.0 g/dL), amylase (28 U/L), sodium (137 mmol/L), potassium (3.4 72 mmol/L), and chloride (99 mmol/L). The urinary test results revealed no signs of infection. Chest and abdominal computed tomography findings were unremarkable, showing no signs of pneumonia or iliopsoas abscesses. Magnetic resonance imaging (MRI) of the left hip revealed low intensity in the femoral head on T2-weighted images (Fig. 1). Joint aspiration was attempted to evaluate possible septic arthritis, but it resulted in a dry tap. Cerebrospinal fluid (CSF) analysis revealed mild pleocytosis (9 cells/mL, with 8 polymorphonuclear cells/mL), markedly elevated protein levels (976.9 mg/dL), and severely decreased glucose levels (1 mg/dL). Gram staining of the CSF revealed gram-positive diplococci, The CSF antigen test was positive for *Streptococcus pneumoniae* and *S. pneumoniae* was subsequently isolated from the CSF culture. The patient received methylprednisolone (500 mg/day), followed by ceftriaxone (4 g/day), vancomycin (2 g/day), and rifampicin (450 mg/day) as an empirical therapy. On day 2, the patient's vision in the right eye deteriorated to light perception. Two sets of blood and CSF cultures were positive for *S. pneumoniae* serotype 23B (determined later at the Department of Bacteriology I, National Institute of Infectious Diseases, Japan). On day 3, vitreous opacity developed in the right eye, which led to blindness. Ophthalmologists assessed the presence of hypopyon, vitreous opacity, phacolysis, and retinal hemorrhage in the right eye (Figs. 2a, 2b). The patient underwent emergency surgery on the right eye, which included vitrectomy. Intraoperatively, the ophthalmologists observed vitreous opacity and judged that bacterial clumps had formed in the vitreous cavity; however, no vitreous specimens were submitted for culture. Therefore, microbiological confirmation of endophthalmitis was not achieved.

On day 4, antimicrobial susceptibility testing revealed that the S. pneumoniae isolate cultured from the cerebrospinal fluid was classified as penicillin-resistant S. pneumoniae (PRSP), with resistance or intermediate susceptibility to almost all β-lactam antibiotics. Susceptibility testing of the blood and cerebrospinal fluid isolates showed non-susceptibility to penicillins, cephalosporins, carbapenems, and macrolides, meeting the definition of multidrug-resistant S. pneumoniae (MDR-S. pneumoniae) (Table 1).

**Table 1:** Antibiotic susceptibility for *Streptococcus pneumoniae*.

Antibiotic	MIC (µg/mL)	Meningitis	Non-meningitis
PenicillinG	1	R	R
Ampicillin	8	NA	NA
Cefotiam	4	R	R
Ceftriaxoneon	1	I	S
Cefditoren-pivoxil	1	I	I
Cefepime	> 2	R	R
Meropenem	1	R	R
Amoxicillin / Clavulanate	4	I	I
Minocycline	4	I	I
Erythromycin	> 2	R	R
Clindamycin	> 1	R	R
Azithromycin	> 4	R	R
Levofloxacin	< 0.25	S	S
Vancomycin	0.5	S	S
Trimethoprim-Sulfamethoxazole	< 0.5	S	S
Chloramphenicol	< 4	S	S
Rifampicin	< 1	S	S

S: susceptible, I: intermediate, R: resistant, MIC: minimum inhibitory concentration.

**Fig. 3:**
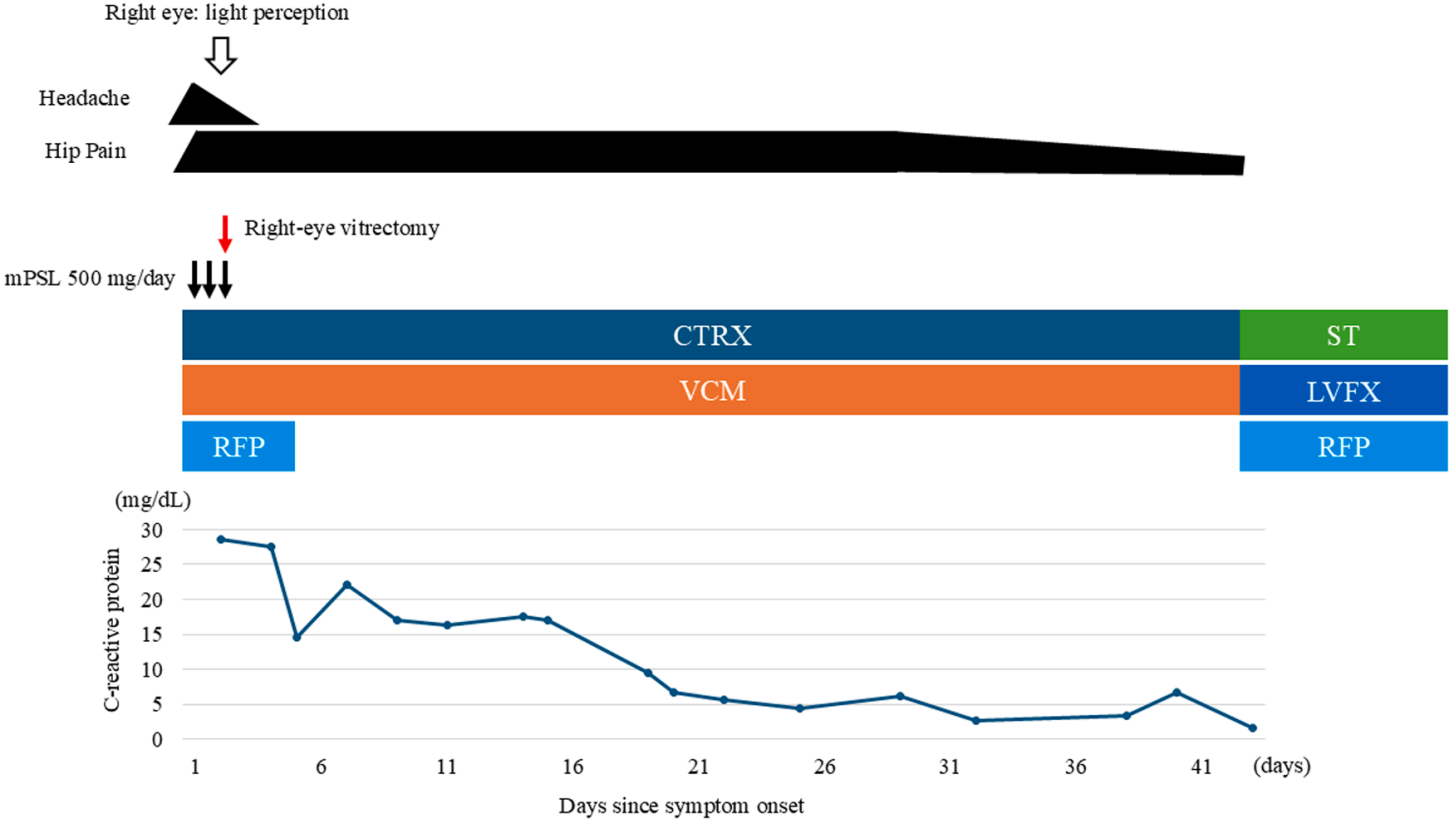
Clinical cours of the patient. The figure summarizes the major clinical events, antimicrobial therapies, and surgical interventions. CTRX: ceftriaxone, LVFX: levofloxacin, RFP: rifampicin, ST: sulfamethoxazole-trimethoprim, VCM: vancomycin.

Based on these results, combination therapy with vancomycin and ceftriaxone at a dosage of 4 g/day was continued, and vancomycin was administered under therapeutic drug monitoring to maintain an AUC/MIC of 400–600 μg·h/mL. On day 10, repeat CSF analysis was performed to assess microbiological improvement because headache and elevated serum C-reactive protein levels persisted. CSF analysis revealed markedly elevated pleocytosis (6575 cells/mL, with 77% polymorphonuclear cells), increased protein levels (263 mg/dL), and decreased glucose levels (11 mg/dL), however, the CSF culture was negative. Although serum C-reactive protein levels improved with ongoing antibiotic therapy, thrombocytosis was also observed. Pain in the right shoulder and hip joints began to decrease on day 30. Intravenous antibiotics were discontinued on day 43, and oral levofloxacin, rifampicin, and trimethoprim/sulfamethoxazole were initiated. Initially, the ophthalmologist anticipated blindness; however, while some constriction of the visual field was observed, the visual acuity of the right eye improved to 0.01 and 0.07 on days 25 and 46, respectively. On day 50, the patient experienced headache, and evaluation by an ophthalmologist revealed that the intraocular pressure in the right eye was elevated to 31.3 mmHg. Consequently, an iridectomy was performed on day 51. The intraocular pressure, measured at 55.0 mmHg the day after surgery, subsequently returned to normal. Although persistent thrombocytosis was observed in previous blood tests, platelet levels were normalized in the blood drawn on day 60. Rehabilitation therapy, including ambulation training, continued. As the CRP levels normalized, antibiotics were discontinued on day 114. On day 123, ophthalmological evaluation revealed a visual acuity of 0.03 and intraocular pressure of 5.7 mmHg in the right eye. The patient was subsequently discharged on day 126. The clinical course of the patients, including antimicrobial treatment, surgical interventions, and major clinical events, is summarized (Fig. 3).

## Discussion

We present a case IPD following splenectomy, presenting as microbiologically confirmed pneumococcal meningitis, clinically diagnosed pneumococcal endophthalmitis, and clinically suspected arthritis. The serotype of *S. pneumoniae* was identified as 23 B.

Because the patient had severe IPD caused by multidrug-resistant PRSP, combination antimicrobial therapy with ceftriaxone, vancomycin and rifampicin was administered. Both meningitis and arthritis improved during treatment.

The use of rifampicin in this case should also be interpreted in relation to the guideline recommendations. The European Society of Clinical Microbiology and Infectious Diseases (ESCMID) guideline [8] state that in regions with a high prevalence of penicillin-resistant pneumococci, vancomycin or rifampicin may be added to a third-generation cephalosporin, such as ceftriaxone or cefotaxime, depending on local pneumococcal susceptibility patterns. In addition, the Infectious Diseases Society of America (IDSA) guideline [9] note that rifampicin should not be used alone because resistance may develop rapidly during monotherapy. Therefore, rifampicin should be used in combination with other antimicrobial agents and considered only when the isolate is susceptible to rifampicin and when the expected clinical or bacteriological response is delayed.

Regarding endophthalmitis, although some constriction of the visual field persisted, surgery successfully prevented the development of blindness. Pneumococcal endophthalmitis is a particularly severe form that poses greater challenges for treatment and carries a significantly higher risk of vision loss, evisceration, and enucleation [10]. Endophthalmitis caused by *S. pneumoniae* has a worse prognosis than that caused by *Klebsiella pneumoniae* or *Pseudomonas aeruginosa* [11]. A study of 38 eyes of 38 patients with pneumococcal endophthalmitis in Taiwan found that the prognosis for visual acuity was poor in all cases, with only six cases (16%) avoiding vision below light perception and ten eyes (26%) undergoing evisceration or enucleation [10]. To improve the prognosis for vision, prompt interventions such as foreign body removal, intraocular antibiotic injection, and vitreous surgery should be performed [10], [12]. Although fluoroquinolones, such as levofloxacin and moxifloxacin, are widely used for the treatment and prophylaxis of endophthalmitis, some *S. pneumoniae* isolates have been reported to exhibit resistance. Therefore, vancomycin is recommended for treating pneumococcal endophthalmitis [10]. Adjunctive corticosteroid therapy in this case should be interpreted with caution. Current guidelines for acute bacterial meningitis recommend dexamethasone before or with the first dose of antimicrobial therapy when pneumococcal meningitis is suspected; the recommended dose is generally 10 mg intravenously every 6 h for 4 days in adults [8], [13], or 0.15 mg/kg every 6 h for 2–4 days [9]. The 2016 UK guidelines also base their clinical recommendations on dexamethasone, although they refer to preclinical evidence that methylprednisolone reduced inflammation in an experimental rabbit model of pneumococcal meningitis [13]. Thus, methylprednisolone pulse therapy is not a standard adjunctive treatment for uncomplicated pneumococcal meningitis.

Nevertheless, several case reports have described the use of high-dose methylprednisolone or pulse-dose glucocorticoids in severe pneumococcal meningitis or meningoencephalitis complicated by inflammatory neurological complications, including cerebral infarction, extensive brain parenchymal lesions, and suspected vasculitic or ischemic changes. In the present case, methylprednisolone pulse therapy was selected based on the comprehensive clinical judgment of neurologists because the patient had severe IPD with pneumococcal meningitis and rapidly progressive visual impairment due to clinically diagnosed endogenous endophthalmitis. This treatment was intended to suppress severe inflammation and preserve visual function rather than serve as standard adjunctive dexamethasone therapy for meningitis.

Although our patient exhibited severe residual visual impairment, the fact that vision did not fall below light perception is believed to be due to early surgical intervention and appropriate antibiotic treatment.

Splenectomy significantly increases the risk of infection because the spleen plays a vital role in orchestrating immune responses, particularly against encapsulated organisms. Notably, susceptibility to infections caused by encapsulated organisms, such as *S. pneumoniae*, *H. influenzae*, and *Neisseria* meningitidis, is markedly increased in individuals with asplenia [14]. This increased susceptibility is primarily due to the loss of the protective functions of the spleen, such as opsonization and phagocytosis, leading to a diminished capacity to effectively combat pathogens [14]. Our patient declined vaccinations, including those for pneumococcal disease, *H. influenzae*, meningococcal disease, and influenza virus, even after splenectomy. We speculate that this refusal to be vaccinated significantly increased the IPD risk.

Serotype 23B pneumococcus has been increasingly isolated worldwide, concurring with the widespread use of pneumococcal vaccines [11], [12], [14]. Notably, serotype 23B is not included in the PCV13, PCV20, or PPSV23 vaccines. During the COVID−19 pandemic, a decline in the incidence of IPD was observed in Switzerland and across Europe. However, as COVID−19 measures eased, IPD incidence increased again in Switzerland, with serotype 23B becoming the predominant strain [15]. In Germany, the isolation rate of pneumococcus has 156 decreased as childhood pneumococcal vaccination has become more widespread; however, the proportion of non-vaccine serotypes, including type 23B, has increased [16]. A pneumococcal carriage survey of children under 5 years of age in Ghana revealed that over 60% of the isolates were non-PCV13 serotypes, with serotype 23B being the most common [17]. Furthermore, serotype 23B has been frequently identified in CSF samples and has emerged as the predominant strain among penicillin-non-susceptible isolates in adult IPD cases [18]. Serotype 23B frequently causes bacteremia with This is a provisional file, not the final typeset article 4 an unknown source and septic arthritis [19], [20]; its infections tend to be more invasive and clinically challenging than those caused by other common nonvaccine-covered serotypes [19], [21]. To the best of our knowledge, there have been no previous reports of endophthalmitis associated with serotype 23B. However, according to a study that analyzed the serotypes of *S. pneumoniae* in ocular infections, serotype 23B was isolated from one case of conjunctivitis [22]. Additionally, this study found that the serotypes of *S. pneumoniae* isolated from ocular infections predominantly belonged to serotypes not included in the pneumococcal vaccine [22]. These findings suggest that serotype 23B S. pneumoniae is often antibiotic-resistant and can cause serious bloodstream and ophthalmological infections and meningitis.

This case report highlights the emergence of penicillin- and third-generation cephalosporin-resistant S. pneumoniae serotype 23B, which presents a unique challenge in patients with simultaneous pneumococcal meningitis, endophthalmitis, and septic arthritis. Although serotype 23B pneumococci are known to cause several infections, to our knowledge, endophthalmitis has not been previously associated with this serotype. Successful treatment outcomes, including patient survival and vision preservation, emphasize the importance of prompt diagnosis, appropriate antibiotic selection, and early surgical interventions.

This case underscores the increasing prevalence of non-vaccine serotypes as pneumococcal vaccination rates increase, emphasizing the need for continued surveillance and vigilance in the management of these infections. However, we already have the PCV21 vaccine, including the 23B serotype. Patients with immunodeficiency, including those with post-splenectomy status, should be considered for vaccination.

## CRediT authorship contribution statement

**Koji Hayashi:** Writing – review & editing, Writing – original draft, Data curation. **Yudai Tanaka:** Writing – original draft, Data curation. **Sayaka Uesaka:** Data curation. **Yuki Abe:** Data curation. **Mamiko Sato:** Data curation. **Yoshihiro Takamura:** Data curation. **Ippei Sakamaki:** Writing – review & editing, Data curation. **Yasutaka Kobayashi:** Data curation. **Yuka Nakaya:** Data curation. **Asuka Suzuki:** Data curation. **Hiroaki Maeda:** Data curation.

## Ethics statement

The patient provided informed consent for the publication of this case report and the accompanying images.

## Funding

This research did not receive any funding.

## Declaration of Competing Interest

The authors declare that they have no known competing financial interests or personal relationships that could have appeared to influence the work reported in this paper.
